# Hi-C technology for detection of chromosomal rearrangements in families with adverse pregnancy outcomes: a preliminary exploratory study

**DOI:** 10.1186/s12884-026-09232-9

**Published:** 2026-05-18

**Authors:** Wei Wang, Yinglu Zhang, Lingna Zhou, Wenyi Chen, Feng Zhang, Bin Yu, Bin Zhang

**Affiliations:** Department of Medical Genetics, Changzhou Maternal and Child Health Care Hospital, Changzhou Medical Center, Nanjing Medical University, No.16 Dingxiang road, Changzhou, 213000 China

**Keywords:** Hi-C technology, Chromosomal rearrangements, Adverse pregnancy outcomes, Optical genome mapping, Structural variant sequencing

## Abstract

Chromosomal abnormalities are a leading cause of adverse pregnancy outcomes. Conventional methods such as karyotyping and chromosomal microarray analysis (CMA) have limited ability to detect cryptic, mosaic, or repeat-region structural variants. This preliminary exploratory study evaluated the feasibility and potential utility of Hi-C in comparison with karyotyping, CMA, optical genome mapping (OGM), and structural variant sequencing (SV-seq) in two families with unexplained or incompletely characterized recurrent adverse pregnancy outcomes. In Family 1, CMA and recurrent abnormal NIPS results suggested maternal mosaic copy number changes, and high depth karyotyping, SV-seq, and Hi-C supported a mosaic unbalanced translocation involving chromosomes 9 and 18; OGM detected the copy number changes but did not resolve the underlying cryptic rearrangement structure. In Family 2, Hi-C, together with OGM and SV-seq, refined the candidate breakpoint regions of a balanced translocation initially detected by karyotyping, and supported transmission of the same rearrangement to the daughter. These findings suggest that Hi-C may serve as a valuable complementary diagnostic tool for identifying cryptic balanced or mosaic chromosomal rearrangements in selected families with unexplained or incompletely characterized recurrent adverse pregnancy outcomes. Larger sample prospective studies are still required to verify its general clinical application in the future.

**Supplementary Information:**

The online version contains supplementary material available at 10.1186/s12884-026-09232-9.

## Introduction

Chromosomal abnormalities are the leading genetic cause of adverse pregnancy outcomes, including recurrent pregnancy loss and intrauterine fetal death, accounting for approximately 60% of clinically unexplained cases [1]. For decades, karyotyping has been the routine diagnostic tool for detecting aneuploidies and large scale rearrangements [2], but it is limited by low resolution (> 10 Mb) and susceptibility to cell culture artifacts. Chromosomal microarray analysis (CMA) improves the detection of submicroscopic copy number variations (CNVs) but, in the context of parental peripheral blood analysis, cannot identify balanced rearrangements (e.g., reciprocal translocations and inversions) and has low sensitivity for low level chromosomal mosaicism [3]. Emerging genomic technologies, including optical genome mapping (OGM) and structural variant sequencing (SV-seq), enable higher resolution of structural variants. However, both methods face technical challenges in highly repetitive genomic regions [4–8].

Hi-C technology, derived from chromosome conformation capture, maps genome-wide chromatin spatial interactions and has been shown to identify complex chromosomal rearrangements that are difficult to resolve by conventional methods [9, 10]. However, clinical evidence supporting its application in reproductive genetics remains limited. Therefore, this preliminary exploratory study aimed to evaluate the performance and potential auxiliary value of Hi-C for detecting cryptic chromosomal rearrangements in two families with unexplained or incompletely characterized adverse pregnancy outcomes, and to compare its detection efficiency with that of conventional and emerging genomic methods.

## Materials and methods

### Patient enrolment

**Fig. 1:**
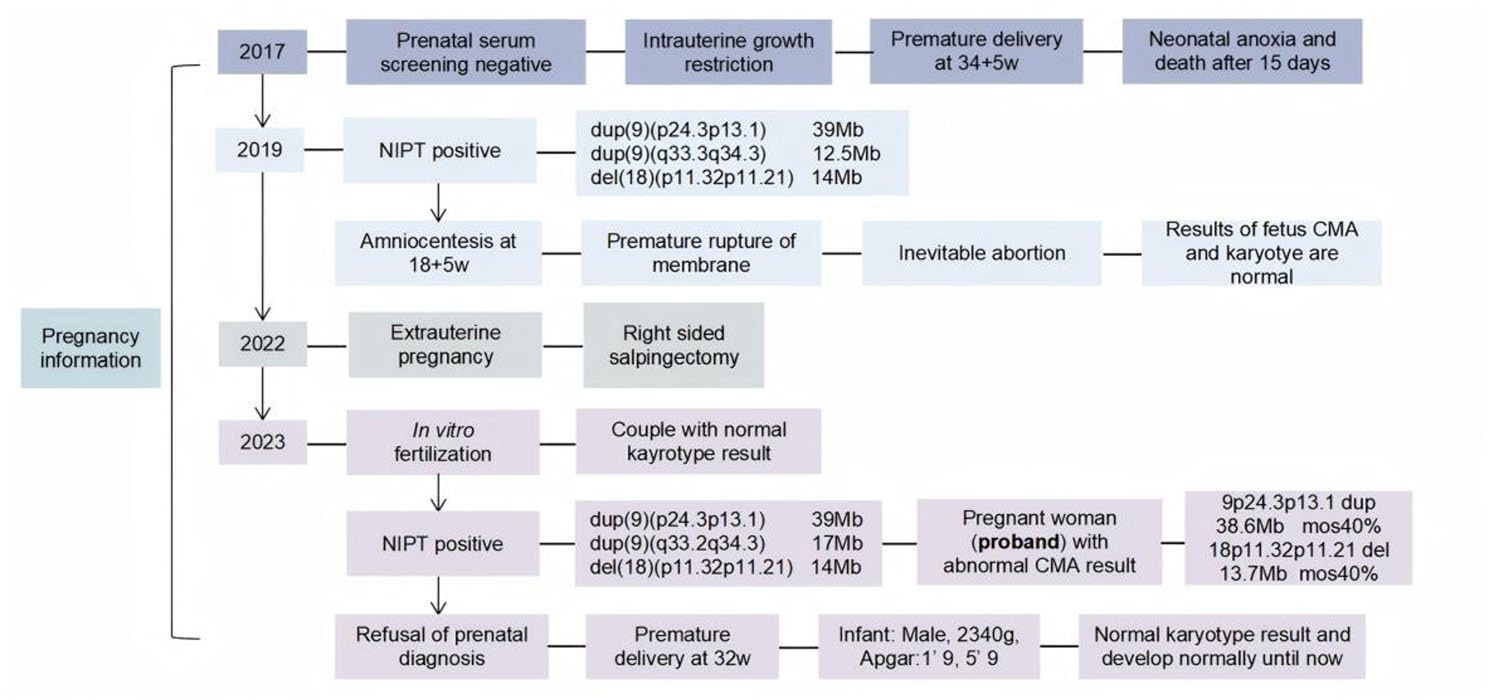
Clinical timeline of family 1

This study enrolled two families with phenotypically healthy members who had experienced adverse pregnancy outcomes and subsequently underwent assisted reproductive technology (ART) procedures at Changzhou Maternal and Child Health Care Hospital in 2024. *Family 1* was a 35-year-old couple with a seven-year reproductive history including one fetal loss at 19 weeks of gestation, one ectopic pregnancy, one neonatal death at 15 days after birth, and one clinically healthy 2 years son. The couple underwent repeated prenatal and genetic evaluations because of unexplained adverse pregnancy outcomes. They received two positive non-invasive prenatal screening (NIPS) results in 2019 and 2023, but prenatal diagnostic tests were negative (Fig. 1). *Family 2* was a 30-year-old couple with a five-year history of recurrent spontaneous abortion (RSA) (Gravida 4, Para 1) and a clinically healthy 4 years old daughter.

Before enrollment, all couples underwent a comprehensive workup for recurrent pregnancy loss, including parental G‑banding karyotyping, female hormonal evaluation (follicle‑stimulating hormone, luteinizing hormone, estradiol, progesterone, prolactin, testosterone), thyroid function and antibody testing, autoimmune screening for antiphospholipid antibodies (anticardiolipin antibody, anti‑β2‑glycoprotein I antibody, lupus anticoagulant), transvaginal uterine imaging, and routine male semen analysis.

Peripheral blood samples were collected from all family members. Advanced genomic analyses such as Hi-C, OGM and SV-seq, were performed on parental peripheral blood samples (proband couples). These analyses aimed to identify cryptic balanced or mosaic structural rearrangements in phenotypically normal couples with unexplained recurrent pregnancy loss.

The study protocol was approved by the Ethics Committee of Changzhou Maternal and Child Health Care Hospital, and informed consent was obtained from all participants. The study complied with the Declaration of Helsinki.

### Noninvasive prenatal screening (NIPS)

NIPS was performed to analyze fetal chromosomal abnormalities using cell-free DNA (cfDNA) isolated from maternal peripheral blood. Maternal plasma was separated via centrifugation, and low-pass whole-genome sequencing (~ 0.1× coverage depth) was conducted on the NextSeq CN500 platform (Berry Genomics, China) [11].

### G-banding analysis

Conventional karyotyping analysis was conducted on the peripheral blood samples collected in heparinised tubes following standard protocols. at a 450-band resolution in accordance with the International System for Human Cytogenomic Nomenclature (ISCN 2020) guidelines.

### Chromosomal microarray analysis (CMA)

CMA was performed on the fetus (amniotic fluid) and the parents (peripheral blood) using a CytoScan 750 K chip (Affymetrix, Santa Clara, CA, USA) following the routine experimental procedure previously described [12]. The raw data were analyzed using Chromosome Analysis Suite (CHAS 4.1) software. Copy number variations (CNVs) were interpreted according to the American College of Medical Genetics and Genomics (ACMG) guidelines.

### Optical genome mapping (OGM)

OGM was performed on the peripheral blood. Ultra-high molecular weight (UHMW) genomic DNA (gDNA) was isolated following the manufacturer’s protocol (Bionano Prep SP Frozen Human Blood DNA Isolation Protocol, Bionano Genomics, San Diego, CA, USA). Subsequently, the direct label and stain (DLS) technique was applied, using direct labeling enzyme 1 (DLE-1) to tag a specific 6-bp motif (CTTAAG) in 750 ng of high-molecular-weight gDNA. The fluorescently labeled DNA molecules were then loaded onto Saphyr chips for linearization and imaging via massively parallel nanochannel arrays. De novo assembly and structural variant (SV) calling were performed using Bionano Access v1.2.1 (Bionano Genomics) and aligned against the Genome Reference Consortium GRCh37. This pipeline detected various variant types, including insertions, deletions, inversion breakpoints, translocation breakpoints, and copy number variations (CNVs) [13].

### Structural variants sequencing (SV-seq)

SV-seq was performed on peripheral blood samples. Genomic DNA was fragmented into 2–5 kb segments, circularized, and enriched via biotinylation. The circularized DNA was then sheared to ~ 500 bp fragments and ligated with sequencing adapters. Library sequencing was conducted on the DNBSEQ-T7 platform (MGI, China). After removing adapter-containing and low-quality reads, high-quality paired-end reads were aligned to the NCBI human reference genome (GRCh37). Data interpretation was performed using the HiSeq platform (Illumina, San Diego, CA, USA).

### Chromosomal structural analysis using Hi-C technology

Hi-C technology is a genome-wide method for detecting pairwise chromatin interactions. Briefly, the Hi-C library was prepared using a standard in situ Hi-C protocol with HindIII digestion, followed by biotinylation, shearing, and streptavidin capture, as previously described [14]. Peripheral blood is collected, and leukocytes are isolated and crosslinked with formaldehyde to preserve protein-DNA interactions. The DNA is then digested with restriction enzymes, and the resulting fragments which adopt a “double-Y” structure are ligated, capturing interactions spanning distances ranging from 100 kb to tens of Mb. The ligated DNA fragment ends are biotinylated, sheared, and enriched using streptavidin-coated beads to isolate the chimeric junctions. This approach enables genome-wide mapping of chromatin interactions.After library preparation, sequencing is performed on the NextSeq 550 platform (Illumina, San Diego, CA). The resulting data are processed and analyzed using the Chromogo software.

## Results

### Family 1 patient description

The detailed pregnancy information for the couple in *Family 1* is presented in Fig. 1. Notably, although the prenatal diagnosis result in 2019 was normal, non‑invasive prenatal screening (NIPS) in 2019 and 2023 both indicated a duplication on chromosome 9 and a deletion on chromosome 18. This abnormal NIPS result may be associated with maternal chromosomal mosaicism, which highlights the high sensitivity of NIPS for detecting maternal genomic alterations. In 2023, karyotype analysis showed normal results for this couple. Subsequently, parental CMA revealed mosaic duplication on chromosome 9 and mosaic deletion on chromosome 18 in the mother.

### Overall results

Husbands in both families showed normal results across all tests. The second karyotype in *Family 1* identified low‑level mosaicism not detected in the initial routine analysis. All results are summarized in Table 1.

**Table 1:** Summary of all analysis results of two families

Family member	Method	CNV duplication			CNV deletion			SV
		Segment	Mosaic (%)	Size (Mb)	Segment	Mosaic (%)	Size (Mb)	
Family 1^+^Proband (female)	Karyotype^*^	NA						46,XX, der(18)t(9,18)(p13;p11.2)[6]/46,XX[90]
	CMA	9p24.3p13.1	40	39	18p11.32p11.21	39	15	NA
	OGM	9p24.3p11.2	50	40	18p11.32p11.2	50	15	Normal
	SV-seq	9p24.3p13.1	50	39	18p11.32p11.1	50	15	Speculate centromeres of 9p13.1 and 18p11.1 have broken and reunion
	Hi-C	9p24.3p23	33	10	18p11.32p11.1	39	15	46, XX, der(18)t(9;18)(p13.1;p11.1)
		9p23p13.1	28	27				
Family 1 Prenatal Diagnosis	Karyotype / CMA	Normal						
Family 2^++^ Proband (female)	Karyotype	NA						46, XX, t(1;19)(p36.3;q13.1)
	CMA	Normal						NA
	OGM	Normal						46, XX, t(1;19)(p36.31;q13.2)
	SV-seq	Normal						46, XX, t(1;19)(p36.31;q13.2)
	Hi-C	Normal						46, XX, t(1;19)(p36.31;q13.2)
Family 2 Daughter	Karyotype^1^ / CMA / SV-seq^2^ / Hi-C^2^	Normal						1.46,XX, t(1;19)(p36.3;q13.1) 2.46,XX, t(1;19)(p36.31;q13.2)

Karyotype^*^: the second time data

^+^NIPS in Family 1 showed recurrent abnormal cfDNA signals involving a chromosome 9 gain and a chromosome 18 loss, whereas fetal diagnostic testing was normal

^++^In Family 2, OGM was not performed on the daughter due to insufficient DNA quality; the proband’s parents underwent Hi-C only (other tests not performed per patient decision)

#### Specific spectrum of Family 1

**Fig. 2:**
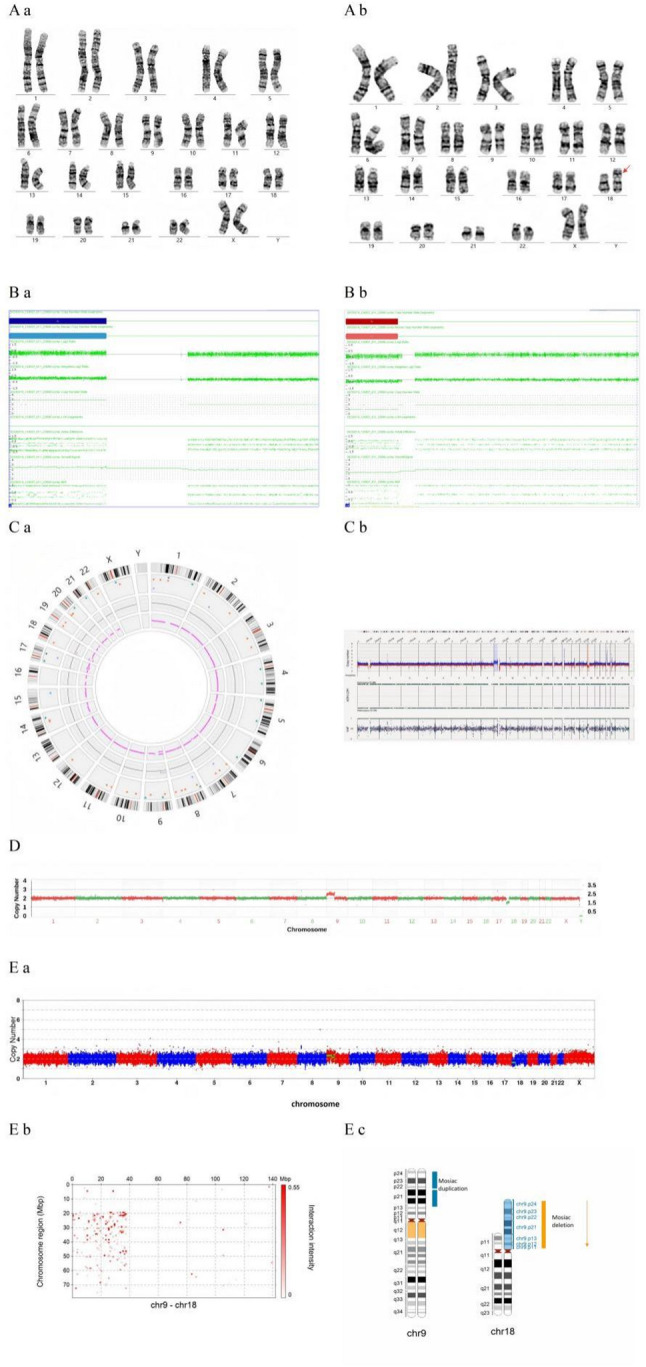
G-banding analysis, CMA, OGM, SV-seq and Hi-C results of proband in Family 1. **A** **a**: The first time karyotype result described as 46,XX. **A b**: The 46,XX, der(18)t(9,18)(p13;p11.2)[6]/46,XX[90]. **B a** and **B b**: CMA signal graph indicating the proband result as arr[GRCH37]9p24.3p13.1(208455_38787480)×3[0.4] (blue bar) and arr[GRCH37]18p11.32p11.21(136228_13825074)×1[0.39] (red bar). **C a**: Circos plot from OGM showing no structural aberrations in proband. **C b**: OGM indicating duplication copy number variants at 9p24.3p11.2 (mos 50%, blue box) and deletion at 18p11.32p11.2 (mos 50%, red box). **D**: SV-Seq showing dup(9) (p24.3p13.1) (mos 50%, red box) and del(18) (p11.32p11.21) (mos 50%, blue box). **E a**. Hi-C presenting dup(9)(p24.3p23) (mos 33%, red box), dup(9)(p23p13.1) (mos 28%, red box) and del(18)(p11.32p11.1)(mos 39%, blue box). **E b**: Hi-C interaction graph between chromosome 9 and 18: X and Y axis presenting translocation interaction region (X axis: from 0 Mb to 140 Mb, Y axis: from 0 Mb to 80 Mb), red spot showing the interaction mainly occur at the short arm of these two chromosome and the darker the color the stronger the interaction effect. **E c**: Schematic representation of the complex rearrangements involving chromosomes 9 and 18. The reciprocal translocation was defined as 46, XX, der(18)t(9;18)(p13.1;p11.1).(orange arrow indicated the chromosomal insertion orientation)

Chromosomal cryptic structural aberrations of Family 1 were successfully identified via the second karyotype analysis, Hi-C, and SV-seq analysis (Fig. 2). The second karyotype was 46,XX,der(18)t(9,18)(p13;p11.2)[6]/46,XX[90]. Initially, routine karyotyping yielded normal results. However, after integrating the proband’s CMA findings, serial NIPS abnormal signals, and clinical pregnancy history, we performed a second round of high‑depth karyotype analysis. This revealed a low‑level mosaic unbalanced translocation: 46, XX, der(18)t(9,18)(p13;p11.2)[6]/46, XX[90] (Fig. 2 A b). This indicated that the proband carried a low-level mosaic unbalanced translocation between chromosome 9 and 18. Among 96 analyzed metaphases, 6 cells carried the derivative chromosome and 90 cells showed a normal female karyotype, corresponding to a mosaic level of approximately 6.3% in cultured lymphocytes. As detailed in Table 1, all technologies identified broadly concordant breakpoints (9p24p13 and 18p11), with mosaic ratios ranging from approximately 30% to 50%. Karyotyping showed a lower mosaic level (6%), likely due to cell culture. The mosaic proportions estimated by different platforms were not directly comparable because karyotyping was based on cultured metaphases, whereas CMA, OGM, SV-seq and Hi-C were performed on bulk peripheral blood DNA and used different analytical assumptions.

For structural aberrations, Hi-C results showed the abnormal trans-chromosomal interaction between chromosome 9 and 18 (Fig. 2 E b and Fig. S1) providing interaction-based evidence supporting the presence of a translocation, consistent with the second karyotype result. SV-seq speculated the centromeres of 9p13.1 and 18p11.1 have broken and reunion, providing additional evidence for the translocation event. OGM showed a negative result for structural variants (Fig. 2 C a and Fig. S2). Of note, the breakpoints are located in highly repetitive regions, where OGM has reduced labeling efficiency.

The chromosome 9 duplication and 18 deletion suggested by NIPS was a false-positive result derived from maternal chromosomal mosaicism, rather than a genuine fetal CNV. The limited resolution of NIPS cannot accurately localize the variant region nor distinguish maternal mosaicism from true fetal abnormalities, leading to an incorrect signal at chromosome 9 and 18. Hi-C provided additional chromatin interaction-based evidence for the rearrangement. Additional detected region of this family is highly repetitive and refractory to fluorescent labeling in OGM, as well as unreliable for sequence alignment in SV‑seq. Hi-C detects rearrangements based on genome-wide chromatin interaction frequencies and is less dependent on fluorescent labeling or short-read alignment. Therefore, it may provide complementary evidence in complex or repetitive genomic regions that are challenging for some conventional or short-read based methods.

The 9p duplication and 18p deletion detected in Family 1 typically involve genes associated with developmental disorders. For instance, heterozygous loss-of-function mutations in *TGIF1* cause holoprosencephaly (OMIM: 602630), and microdeletions of 18p11.3 encompassing *TGIF1* have been reported in patients with intellectual disability and growth retardation [15–17]. However, the proband in our study was phenotypically normal, which may be attributed to the mosaicism.

**Fig. 3:**
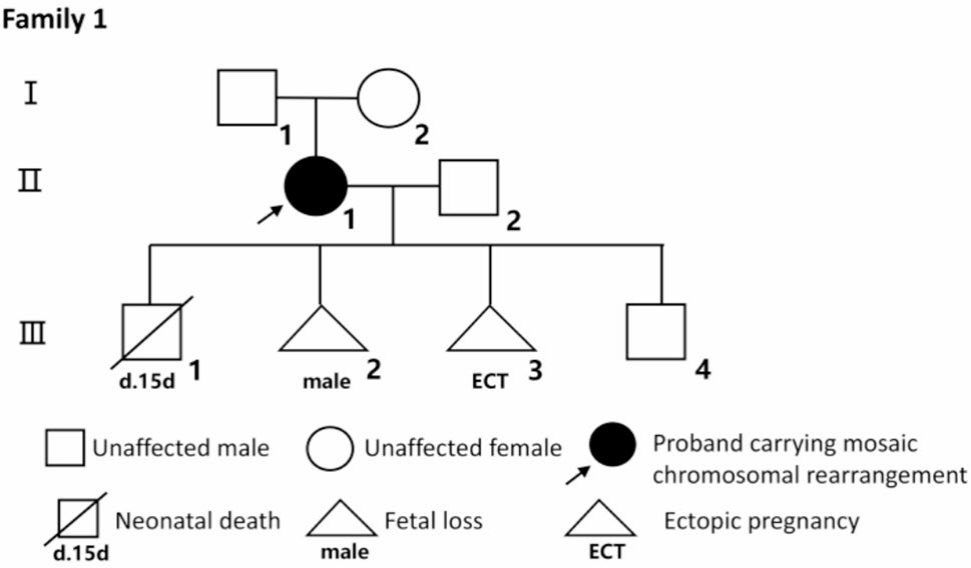
Pedigree of Family 1. The proband (II-1) carried a somatic mosaic unbalanced translocation der(18)t(9;18)(p13.1;p11.1); II-1 had three pregnancy losses: one fetal loss at 19 weeks, one ectopic pregnancy, and one neonatal death at 15 days. All other family members showed normal genetic results

The proband was phenotypically healthy (college-educated, no clinical abnormalities) and carried a low level mosaic unbalanced translocation. Her parents and young son showed normal phenotypes, and their Hi‑C results were negative (Fig. 3). The proband’s mother had no history of miscarriage and had only one child (the proband). Her son was evaluated by a pediatrician and had normal growth and development, with negative karyotyping and Hi‑C results. Collectively, these findings supported that the mosaic rearrangement is a sporadic somatic event without familial inheritance, explaining why the proband and her offspring do not exhibit the clinical phenotypes typically associated with 9p trisomy or 18p monosomy.

#### Specific spectrum of Family 2

**Fig. 4:**
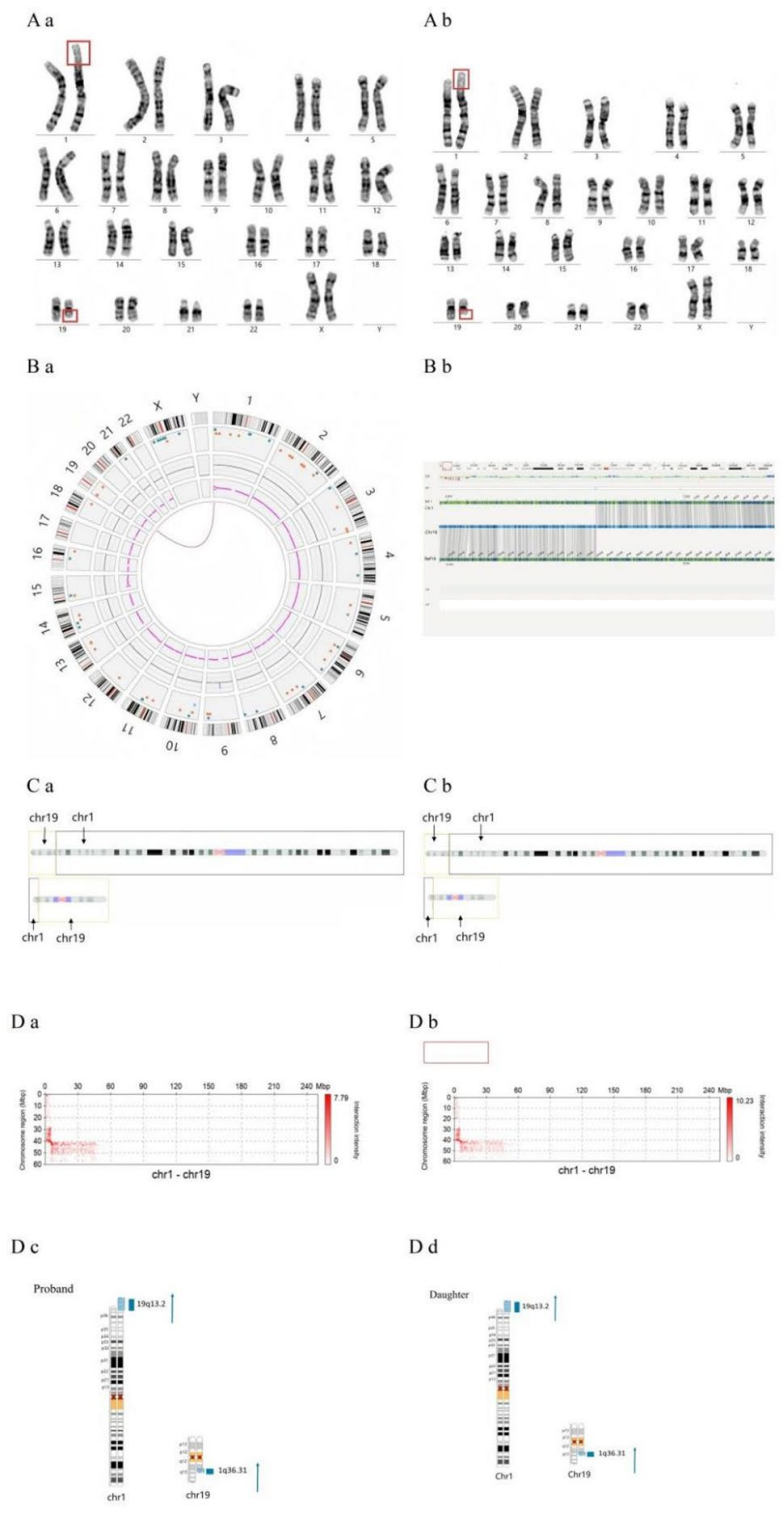
G-banding analysis, CMA, OGM, SV-seq and Hi-C results of proband and her daughter in Family 2. **A a**: The karyotype of proband is 46,XX, t(1;19)(p36.3;q13.1). **A b**: Her daughter has inherited the same karyotype. **B a** and **B b**: OGM indicating the proband’s translocation breakpoints at 1p36.31 and 19q13.2. **C a** and **C b**: SV-Seq showing the same breakpoints at 1p36.31 and 19q13.2 of both proband (a) and her daughter (b). **D** **a** (proband) and **D** **b** (daughter): Hi-C interaction graph between chromosome 1 and 19, X and Y axis presenting balanced translocation interaction region. **D c** (proband) and D d (daughter): The Hi-C speculated scheme of family 2 at the same breakpoints at 1p36.31 and 19q13.2. (Blue arrow showed the junction orientation)

Family 2 showed a balanced translocation between chromosomes 1 and 19 in the female proband, and the same rearrangement was detected in her clinically healthy daughter, indicating vertical transmission from the proband to her daughter (Fig. 4). As summarized in Table 1, initial karyotyping estimated the breakpoints at 1p36.3 and 19q13.1, while CMA yielded negative results for all family members. Subsequent high-resolution genomic techniques, including OGM, Hi-C, and SV-seq, refined the breakpoints to 1p36.31 and 19q13.2, confirming the translocation. Notably, the rearrangement exhibited an inverted orientation compared to conventional translocations (Fig. 4). It is important to highlight that OGM analysis was performed only on the proband’s couples within Family 2 due to limited high-molecular-weight DNA availability from daughter’s sample and other Products of Conception (POC). The proband’s parents declined additional genetic testing beyond Hi‑C, which was performed solely to determine the origin of the translocation.

**Fig. 5:**
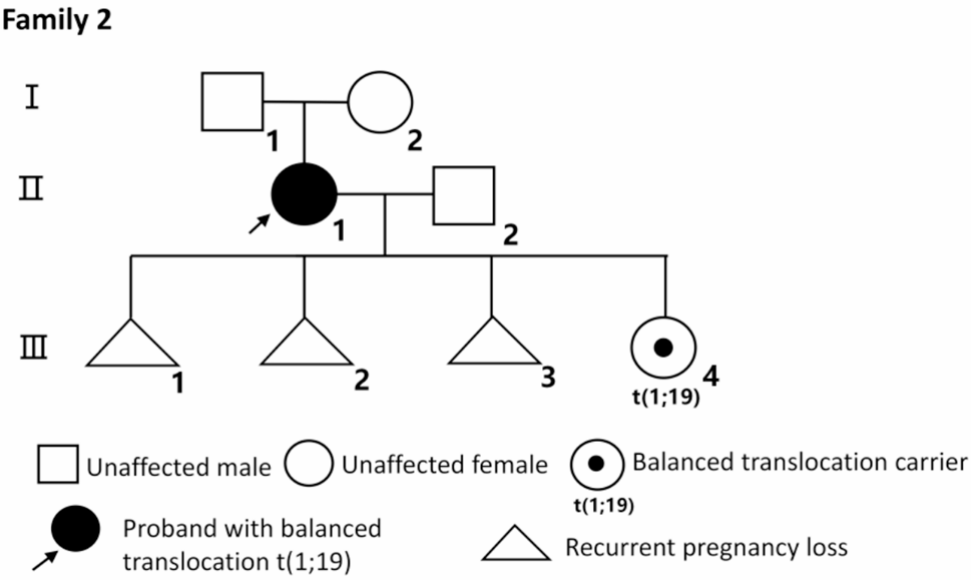
Pedigree of Family 2 with balanced translocation t(1;19)(p36.31;q13.2). The proband (II-1, arrow) had three previous recurrent pregnancy loss (gestational ages unclear, cause unknown). Her daughter (III-4) is a balanced translocation carrier. Additional parental Hi-C analyses supported a *de novo* origin of the balanced translocation in the proband

Although karyotyping identified the balanced translocation, Hi-C provided additional interaction based evidence and confirmed the identical rearrangement structure and breakpoints in both the proband and her daughter (Fig. 5). This high-precision information supports accurate genetic counseling and PGT-SR design. To explore the origin of the balanced translocation, we further performed Hi-C analysis on the proband’s parents. Hi-C analysis of the proband’s parents did not detect the same interchromosomal interaction signal in peripheral blood, suggesting an apparently d*e novo* origin in the proband and was subsequently transmitted vertically to her daughter. However, parental germline mosaicism cannot be completely excluded.

## Discussion

This study was conducted as a preliminary exploratory analysis of two families with recurrent adverse pregnancy outcomes. The aim was to explore the application characteristics of multiple cytogenetic and genomic detection platforms in complex or cryptic chromosomal rearrangements. Given the limited sample size, the findings are observational and descriptive rather than confirmatory.

In this exploratory cohort, we evaluated utility of Hi-C, OGM, SV-seq and conventional genetic detection methods in challenging structural variation cases [18]. Conventional G-banding karyotyping is limited by its relatively low resolution and may be affected by cell culture-related artifacts [19]. CMA is useful for detecting copy-number changes but cannot identify balanced chromosomal rearrangements, which limits its application in some reproductive genetic scenarios [20]. In the present study, Hi-C showed complementary value in selected cases by providing chromatin interaction based evidence for rearrangements involving regions that were difficult to resolve using conventional approaches [1, 20].

OGM is an emerging technology with potential for detecting structural variants. However, its performance may be affected in complex or repetitive genomic regions when fluorescent labeling is sparse or unevenly distributed [20]. This limitation was reflected in Family 1, in which OGM did not detect the cryptic translocation in highly repetitive region. By contrast, SV-seq is relatively cost-effective for screening chromosomal rearrangements and copy number variations, but it may have limited ability to resolve breakpoints located in centromeric or highly repetitive regions [1, 7]. In this comparative analysis, Hi-C provided interaction-based evidence supporting cryptic rearrangements and helped refine candidate breakpoint regions, particularly in genomic regions that were difficult to characterize using other methods. In Family 2, karyotyping identified a balanced translocation, and Hi-C provided additional interaction-based evidence involving the same chromosomal regions and helped refine the candidate breakpoint regions. These Hi-C inferred regions should be interpreted as candidate breakpoint regions rather than nucleotide-level breakpoints.

In addition to variant detection performance, the clinical applicability of different genetic platforms should be considered in relation to cost, turnaround time, and technical requirements [21]. Conventional G-banding karyotyping and CMA remain widely used in routine clinical practice because of their relatively established workflows and accessibility [22]. OGM and SV-seq may provide additional information for structural variant detection, but their performance can vary according to sample quality, genomic context, and analytical strategy [23, 24]. Compared with these methods, Hi-C requires more complex library preparation and dedicated bioinformatic analysis [25–28]. Although Hi-C may provide complementary information for cryptic or complex rearrangements in selected cases [29], its longer turnaround time and higher technical requirements may limit its current use as a routine first-line clinical test [30]. Such practical distinctions should be fully considered in clinical decision making rather than focusing solely on diagnostic performance.

The abnormal NIPS result in Family 1 illustrates that maternal copy number changes or mosaic chromosomal rearrangements may interfere with prenatal screening interpretation [31]. Mosaic translocation is a rare cytogenetic finding, and standardized criteria for pathogenic interpretation remain limited [32]. In the present families, maternal chromosomal rearrangements and adverse pregnancy histories were observed simultaneously. However, a direct causal relationship could not be established. Because informative fetal or embryonic genetic data were unavailable, we could not determine whether the maternal structural rearrangements directly contributed to the recurrent pregnancy complications [33, 34]. Variable pregnancy outcomes may be related to the level of mosaicism and random chromosomal segregation. Embryos inheriting an unbalanced derivative chromosome may be at increased risk of miscarriage or developmental abnormalities, but this possibility requires direct embryonic or prenatal genetic evidence.

The somatic mosaicism identified in Family 1 may have originated from post-zygotic mitotic errors during early embryonic development, based on the absence of this mosaic variant in peripheral blood samples from other family members [33, 34]. However, this interpretation remains speculative and cannot replace direct experimental validation. Therefore, for Family 1, the available evidence supports careful reproductive counseling and prenatal diagnosis in future pregnancies, rather than routine recommendation of PGT-SR. The possibility of germline mosaicism should also be discussed with the couple.

For Family 2, the detection of the same balanced reciprocal translocation in the daughter by karyotyping supported vertical transmission from the carrier mother. In this context, PGT-SR or prenatal diagnosis should be discussed, because carriers of balanced reciprocal translocations may produce unbalanced gametes.

One case harbored a partial 18p deletion covering the *TGIF1* locus. Although previous studies have reported that variants within this region are related to developmental disorders [35–37], it remains inappropriate to correlate this chromosomal segmental imbalance with individual adverse pregnancy outcomes in this limited cohort [38]. The gene annotation is therefore provided for descriptive purposes only and should not be interpreted as evidence of a mechanistic or causal relationship with the observed pregnancy outcomes.

Several limitations of this study should be acknowledged. First, the small sample size lacks statistical power for formal inference and may introduce sampling bias. Second, severely degraded products of conception samples failed to meet high-throughput sequencing quality requirements, resulting in a lack of informative embryonic genetic evidence. Third, orthogonal validation methods, such as FISH, long-read sequencing, or breakpoint-spanning PCR, were not performed. Therefore, nucleotide-level breakpoint resolution and the detailed structural configuration of the rearrangements could not be independently validated. Fourth, the experimental design was not fully uniform across families, and differences in sample availability and sample quality limited direct comparison among platforms. For example, OGM data were unavailable for the daughter in Family 2, and multi-platform testing was not performed in all available family members. These factors limited the completeness of inter-platform comparison and should be considered when interpreting the findings. Finally, the current clinical use of Hi-C remains constrained by cost, experimental complexity, turnaround time, and bioinformatic requirements.

In the future, we will continue to expand the sample size in subsequent studies, adopt unified multi-platform detection procedures, and introduce multiple orthogonal verification methods. Prospective standardized collection of embryonic and conception specimens will also be strengthened to provide more sufficient genetic evidence for exploring clinical correlation.

## Conclusion

In summary, this preliminary exploratory study describes the use of several genetic testing platforms in two families with complex chromosomal rearrangements. Hi-C may serve as a complementary parental testing approach in selected couples with unexplained or incompletely characterized recurrent adverse pregnancy outcomes. It may be useful for identifying cryptic balanced or mosaic structural rearrangements in parental blood samples. OGM and SV-seq also have distinct advantages and limitations depending on the genomic context and clinical question. Given the limited sample size, non-uniform testing strategy, and absence of orthogonal breakpoint validation, these findings should be interpreted cautiously. Larger prospective studies are needed before Hi-C can be recommended for routine clinical use.

## Supplementary Information

Supplementary Material 1.

## Data Availability

Data are located in controlled access data storage at Changzhou Maternal and Child Health Care Hospital.The datasets generated and analyzed during the current study are not publicly available due to reasons of sensitivity in China but are available from the corresponding author on reasonable request.
