# Supplementary material for: Hi-C technology for detection of chromosomal rearrangements in families with adverse pregnancy outcomes: a preliminary exploratory study
Source: BMC Pregnancy Childbirth. 2026 May 18;26:740. doi: 10.1186/s12884-026-09232-9 (PMC13348099; doi:10.1186/s12884-026-09232-9)
Supplement: Supplementary file 1 — Supplementary Material 1. [file 12884_2026_9232_MOESM1_ESM.docx]

**Supplementary Materials**

| Y axis:chromosome 18 | X axis:chromosome 9  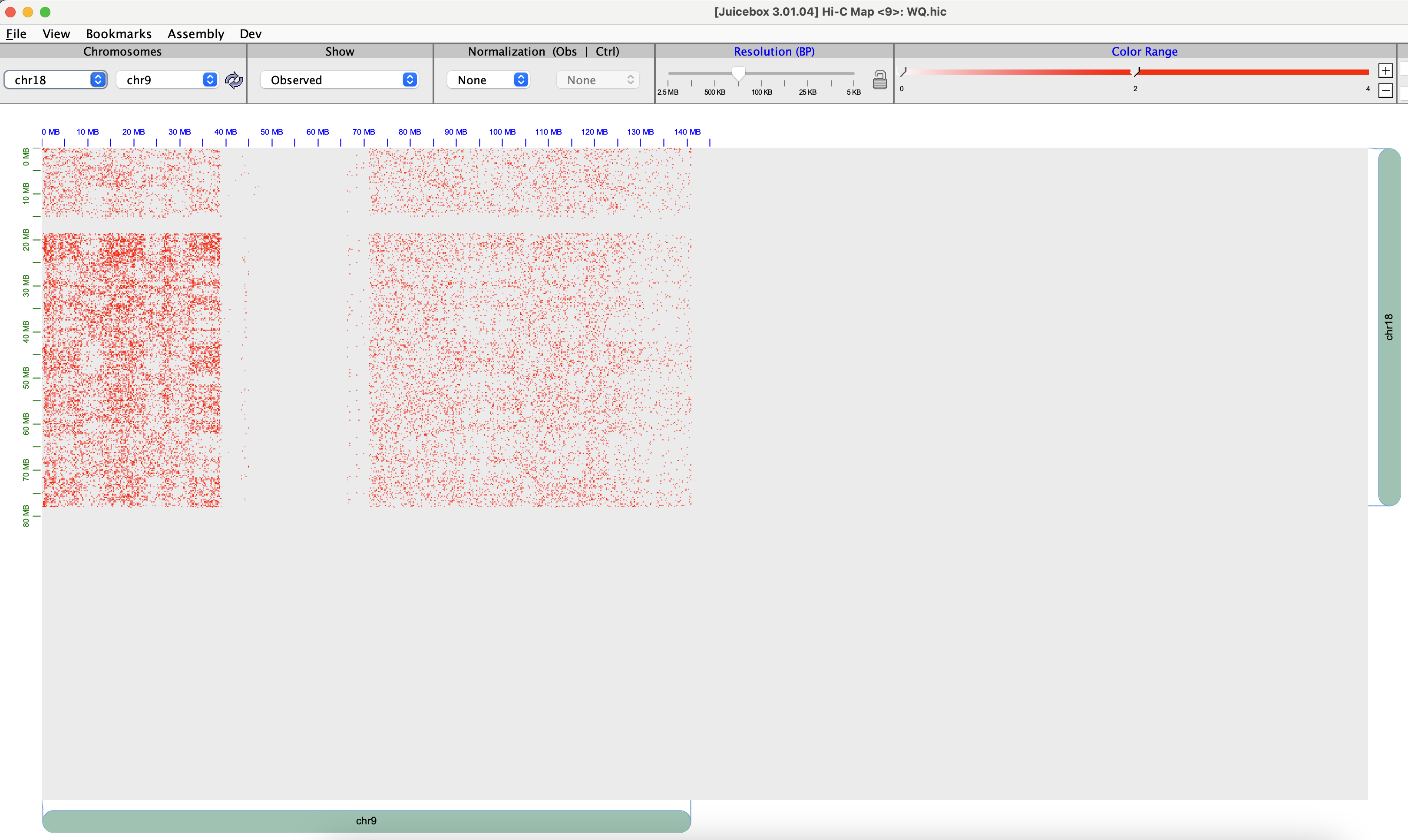 | Contact count | High  ↑  Low |
| --- | --- | --- | --- |

**Supplementary Figure S1. Hi-C interaction heatmap between chromosome 9 and 18 in the proband of Family 1.** The clear inter-chromosomal contact hotspot precisely identifies the translocation breakpoint of der(18)t(9;18)(p13.1;p11.1), which was not detected by OGM.

| Chromosome 9 |
| --- |
| 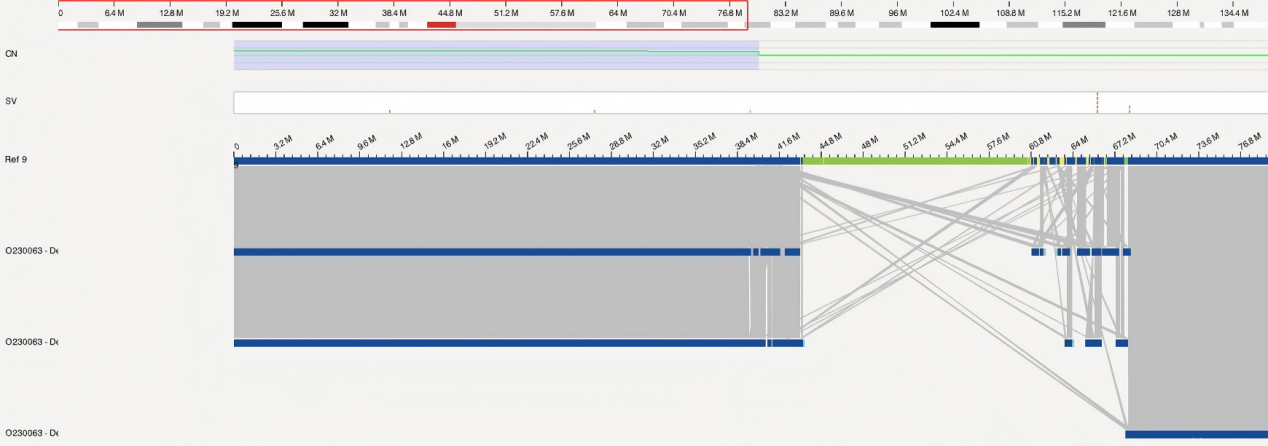 |
| Chromosome 18 |
| 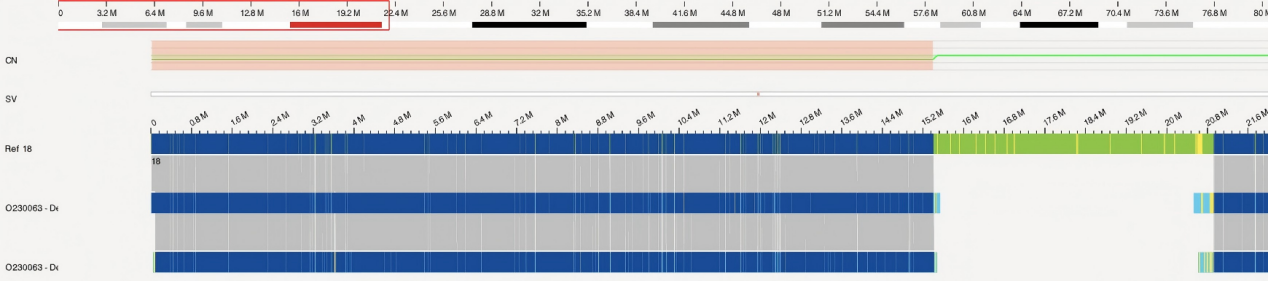 |

**Supplementary Figure S2. OGM barcode maps of chromosome 9 and 18 in the Family 1 proband.** OGM identified mosaic copy number changes but did not detect the cryptic unbalanced rearrangement.
